# Supplementary figures and images for: CRISPR/Cas9-loaded stealth liposomes effectively cleared established HPV16-driven tumours in syngeneic mice
Source: PLoS One. 2021 Jan 7;16(1):e0223288. doi: 10.1371/journal.pone.0223288 (PMC7790238; doi:10.1371/journal.pone.0223288)

### Figure 3C HMGB-1

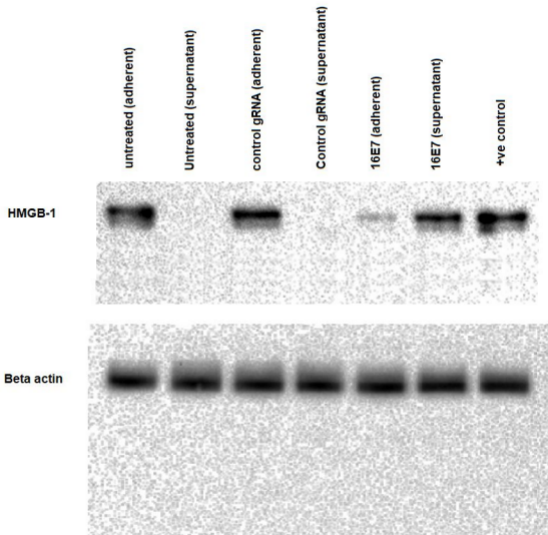

**Fig 1C Rb and loading control**

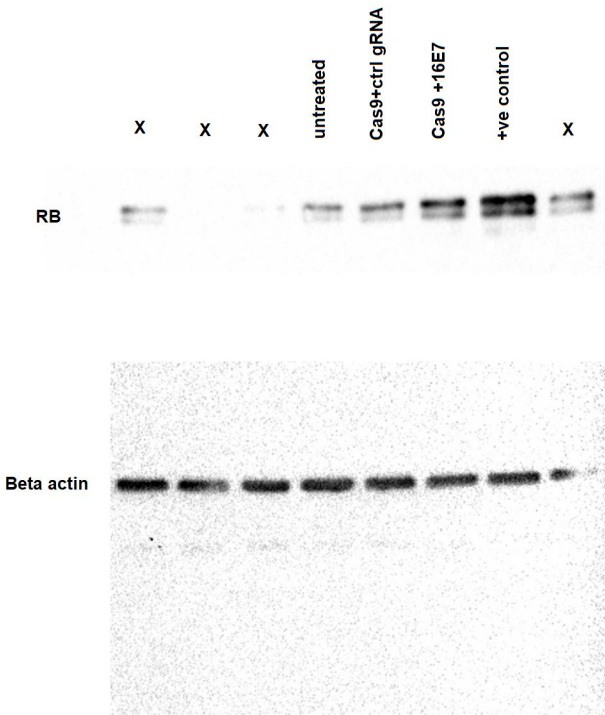

Fig 1D T7E1 assay

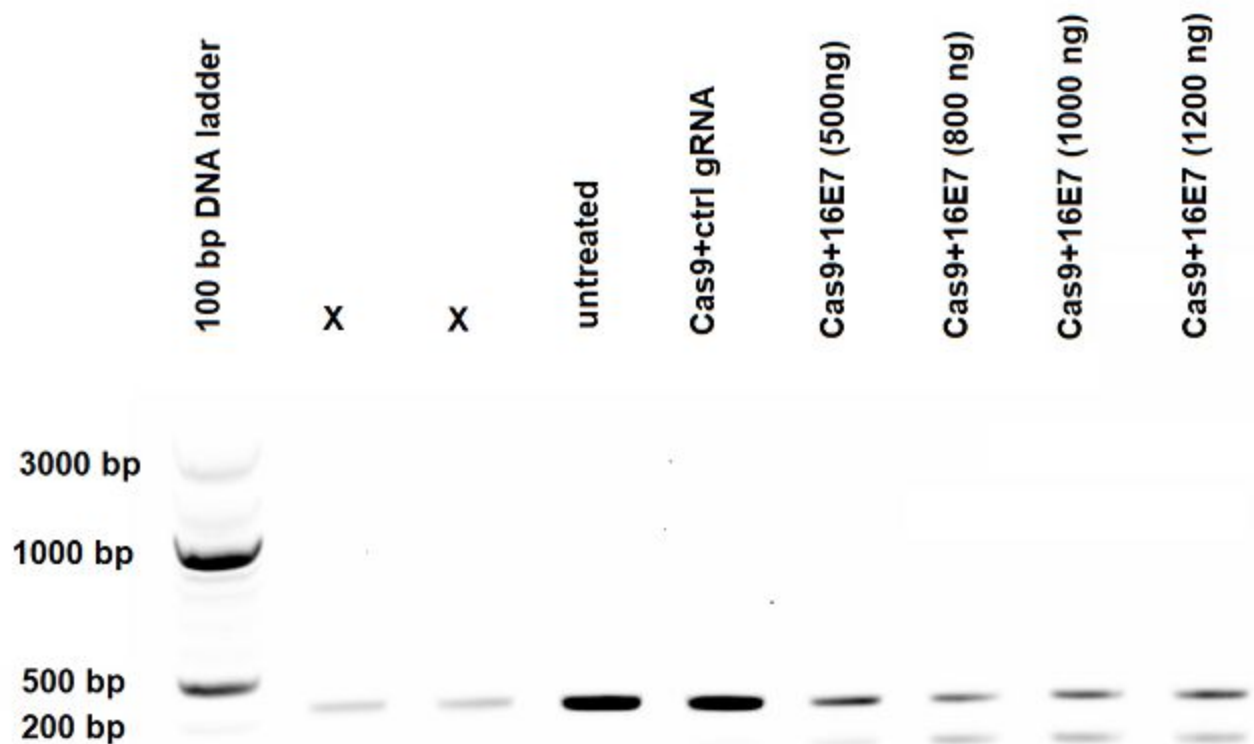

Supplement: S1 Raw images — (PDF) [file pone.0223288.s001.pdf]
